# Online training to improve BLS performance with dispatcher assistance? Results of a cluster-randomised controlled simulation trial

**DOI:** 10.1186/s13049-024-01226-w

**Published:** 2024-06-04

**Authors:** Janina Bathe, Anne Daubmann, Christoph Doehn, Antonia Napp, Marco Raudies, Stefanie Beck

**Affiliations:** 1 Centre of Anaesthesiology and Intensive Care Medicine, Hamburg-Eppendorf University Medical Centre, Hamburg, Germany; 2 Department of Medical Biometry and Epidemiology, Hamburg-Eppendorf University Medical Centre, Hamburg, Germany; 3 Hamburg Fire Brigade, Hamburg, Germany

## Abstract

**Background:**

The prognosis for patients improves significantly with effective cardiopulmonary resuscitation (CPR) performed by bystanders. Current research indicates that individuals who receive CPR from trained bystanders have a greater likelihood of survival compared to those who receive dispatcher-assisted CPR from untrained laypersons. This cluster-randomised controlled trial assessed the impact of a 30-min online training session prior to a simulated cardiac arrest situation with dispatcher-assisted CPR (DA-CPR) on enhancing Basic Life Support (BLS) performance.

**Methods:**

This study was performed in 2018 in Hamburg, Germany. The primary outcome was the practical BLS skills of high school students in simulated out-of-hospital cardiac arrest scenarios with dispatcher assistance. The intervention group participants underwent a 30-min online BLS training session, while the control group did not receive an intervention. It was hypothesized that the average practical BLS scores of the intervention group would be 1.5 points higher than those of the control group.

**Results:**

BLS assessments of 286 students of 16 different classes were analysed. The estimated mean BLS score in the intervention group was 7.60 points (95% CI: 6.76 to 8.44) compared to 6.81 (95% CI: 5.97 to 7.65) in the control group adjusted for *BLS training* and *class*. Therefore, the estimated mean difference between the groups was 0.79 (95% CI: -0.40 to 1.97) and not significantly different (*p*-value: 0.176). Based on a logistic regression analysis the intervention had only a significant effect on the chance to pass the item “vertically above the chest” (OR = 4.99; 95% CI: 1.46 to 17.12) adjusted for *BLS training* and *class*.

**Conclusion:**

Prior online training exhibits beneficial impacts on the BLS performance of bystanders during DA-CPR. To maximise the effect size, online training should be incorporated into a set of interventions that are mutually complementary and specifically designed for the target participants.

**Trial registration:**

DRKS00033531. "Kann online Training Laien darauf vorbereiten Reanimationsmaßnahmen unter Anleitung der Leitstelle adäquat durchzuführen? " Registered on January 29, 2024.

## Introduction

Early provision of cardiopulmonary resuscitation (CPR) by bystanders can increase the chance of survival two- to four-fold, reduce the risk for morbidities and improve neurological outcome in out-of-hospital cardiac arrest [1, 2]. Comprehensive training of the population and dispatcher assistance via telephone during the emergency call (DA-CPR) are effective to improve bystander CPR-rates and the chance of survival [3–8]. The implementation of comprehensive Basic Life Support (BLS) training for the population is arduous and even the recommended training of children in schools (‘kids save lives’ program) is implemented only fragmentarily. Lack of trainers, equipment and perceived need for training, as well as unsolved funding are the main reasons for the moderate implementation rate [9–11]. Dispatcher assisted telephone CPR is well established in many countries but the capability to recognise a cardiac arrest quickly and accurately varies remarkably between the different dispatch centres [12]. The evidence favours dispatcher assisted bystander- CPR compared to no bystander CPR, but there are many unsolved issues as to optimal instruction sequence and the use of key words. [13, 14] Video calls hold promise in providing visual support to bystanders and supplying dispatchers with visual information from the scene to enhance instructions and feedback. The technology however needs to be further developed and fully implemented [15, 16]. Current observational studies suggest that victims who received CPR by trained bystanders have better survival chances compared to those who received dispatcher-assisted bystander CPR by untrained bystanders [17].

BLS training for the general public is a crucial component in a system aimed at maximizing survival chances. Alternative training methods, such as self-directed digital learning, are recognized as being as effective as traditional instructor-led practical BLS trainings. These methods are highly recommended due to their greater accessibility [18].

This study investigated whether a combination of self-directed learning through an online course and dispatcher assistance could enhance the BLS performance of laypersons. The hypothesis was that individuals who had undergone online training would outperform those without prior training in a simulated BLS scenario with dispatcher assistance. The primary outcome was the mean scores of the practical BLS assessment. Secondary outcomes included pass rates at the item level and for the entire exam.

## Methods

### Trial design

This cluster-randomised, rater-blinded controlled study was conducted with high school students at the Hamburg-Eppendorf University Medical Centre in September 2018.Students in grades eleven and twelve (expected age 16–18 years) were randomly selected to perform BLS with the help of a dispatcher, either without intervention (control group) or after undergoing a 30-min online BLS training session (intervention group). The BLS performance of the students was assessed with a simulated dispatcher assisted out-of-hospital cardiac arrest scenario. This assessment was incorporated into a special neuroscience event at the University Hospital, which served as the incentive to participate in the study. Following their evaluation, all students were provided with feedback and an additional instructor-led practical BLS training.

### Participants

The students were recruited via newsletter addressed to all high school biology teachers of Hamburg. Students and teachers were invited to an event with lectures on neuroscientific topics.

All students, along with their parents or legal guardians, were provided with written information about the objective of the study. Inclusion criteria were voluntary participation and written informed consent to participate on hand. Exclusion criteria was any mental or physical inability to perform CPR.

### Randomisation and blinding

The students were systematically randomised on a class-by-class basis into either the intervention or control group, alternating at a 1:1 ratio. This was done following the sequence of the participant list provided by the teachers during a preparatory meeting for the event. Students of one class represented one cluster. Only the participants of the intervention group were provided with the link to the interventional training and were asked not to pass on the information. The assessors were blinded for the group affiliation of the students.

### Intervention

**Fig. 1:**
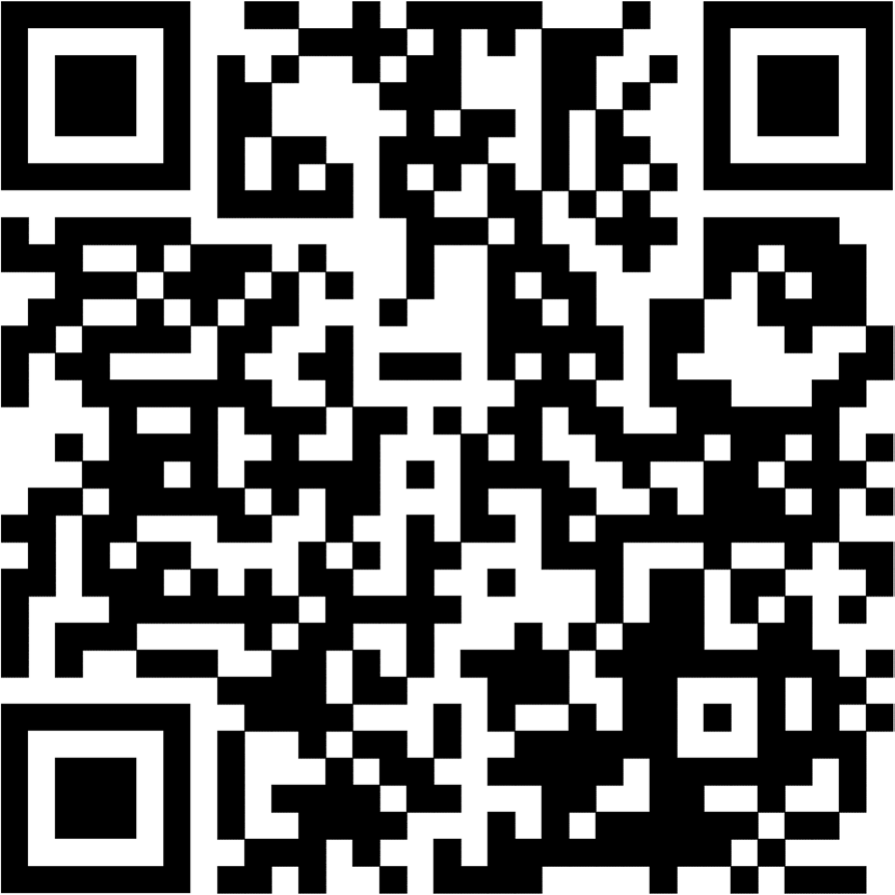
QR-link to the online training platform

The students assigned to the intervention arm prepared for the assessment using an interactive website on cardiac arrest, endorsed by the Hamburg Open Online University (https://etraining.uke.de/HOOU/rea/free.php). Teachers were instructed to allocate half an hour of self-study time for the students during biology lessons in the two weeks leading up to the assessment. The students were tasked with working through the text and interactive tasks of the modules “Warum handeln?” (“Why act?”) and “Was tun?” (“What to do?”). The website provides fundamental information on the physiology and implications of a cardiac arrest, as well as the procedures of BLS (link via QR-Code: Fig. 1). It includes interactive elements and videos demonstrating how to perform effective chest compressions and the components of an effective chain of survival.

The control group had no special CPR training before the scenario testing of BLS skills. They continued with the topics in biology that were currently on the curriculum.

### Assessment

The assessment was structured around a three-minute, single-rescuer cardiac arrest scenario involving an unresponsive individual lying on the school floor. A written summary of the scenario was posted on the front door of each assessment room, and the assessments commenced with a synchronized auditory signal. The Pre-recorded dispatcher-assisted telephone CPR instructions, adhering to the algorithm of the Emergency Medical Service of the Hamburg Fire Department, was initiated when the student made the emergency call, or automatically after 45 s at the latest. The instructions are for chest-compression only and included two closed questions confirming cardiac arrest. For the assessment Practiman® (Vimetecsa™, Alicante) manikins and a structured rating checklist with 10 binary items were used (information about validity and reliability of the assessment instrument can be extracted from this publication) [19]. The whole BLS exam was passed when all items were rated “passed”. The students were assessed by medical students who were trained to use the rating checklist and blinded for the group affiliation of the students.

### Endpoints

Primary endpoint were practical BLS skills of the students. To measure this, the mean of passed items was compared between the groups. Secondary endpoints were pass-rates for the whole BLS exam and every item of the BLS assessment.

### Sample size calculation

Sample size calculation was based on previous studies. In Süss-Haveman et al. 2020 we observed a decrease of practical BLS skills of 0.76 points within one year after training [20]. As an active intervention we aimed at doubling the effect in the positive direction as effective and calculated with a mean difference in the BLS score of 1.5 points. With a standard deviation of 3 points, an alpha of 0.05 (two-sided hypothesis), an average cluster size of 20 students per class with an intra-cluster correlation (ICC) of 0.11 we needed 200 students per arm to achieve a power of 80%. Therefore, 10 clusters with 20 students each were needed to detect the assumed difference between intervention and control group. It was expected that the students of the control would perform worse. All teachers, along with their respective classes, who participated in the second preparatory meeting were included in the study.

### Statistics

Descriptive statistics were evaluated for all randomised students by group. The absolute and relative frequencies were calculated for the categorical values. Means and standard deviation (SD) were determined for continuous variables**.** For the statistical analysis of the primary outcome, we used a mixed model with number of points in the BLS exam as endpoint and group (intervention vs. control), BLS-training status as fixed effects, and class as a random effect. Means and corresponding 95% confidence intervals (CI) were reported. For the binary endpoints (pass-rates for the whole BLS exam and every item), a mixed logistic regression was performed with the same specifications as in the model for continuous endpoints. Odds ratios and corresponding 95% CI were reported. Two-sided *p*-values < 0.05 were considered as significant. The analyses were performed by a statistician of the Department of Medical Biometry and Epidemiology of the Hamburg-Eppendorf University Medical Centre using SPSS, version 24 (IBM Corp, Armonk, NY, USA).

## Results

### Participants

**Fig. 2:**
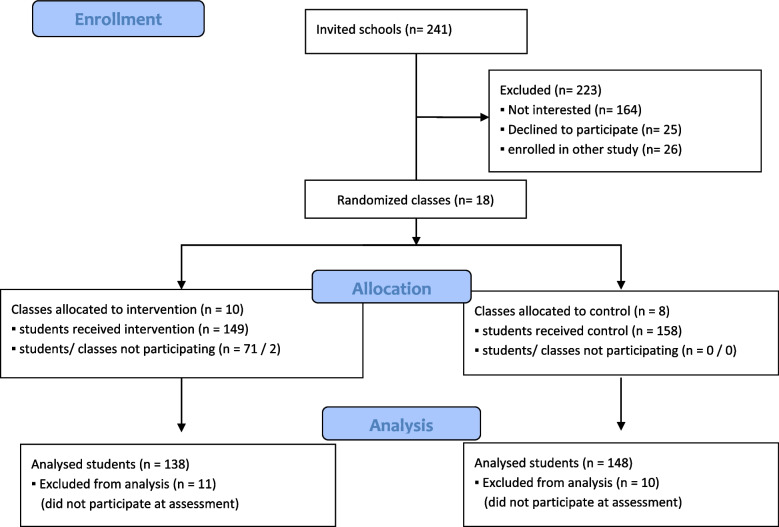
Flow Chart of the participating high school students

Two hundred twenty senior high school students were randomised to the intervention and 158 to the control group. In both the intervention and control groups, there were students who, despite being randomised, did not participate in the event and therefore could not be assessed. 148 assessments of students of the intervention and 138 of the control group students were analysed after exclusion (Fig. 2). Demographic data but not BLS training status were comparable between the groups. Data are presented in Table 1.

**Table 1:** Demographic data of the participants

	**Intervention**	**Control**
Age - Years (SD)	17.4 (0.8)	17.8 (1.2)
Gender—% Male (NO.)	46.9 (69/ 137)	39.6 (53/134)
High – Cm(SD)	173.6 (10.1)	173.1 (11.5)
Weight – Kg (SD)	66.5 (13.1)	66.6 (14.6)
Never Bls-Training—% (NO.)	20.7 (30/ 145)	27.1 (36/133)
BTraining< 1 Year—% (NO.)	28.3 (41/ 145)	35.3 (47/133)

### Primary outcome

**Fig. 3:**
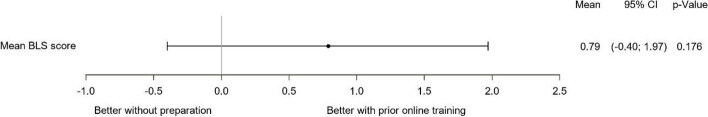
The mean difference in the BLS score between the groups is 0.79 (95% CI: -0.40 to 1.97) favouring performance with prior online training. The difference is not significantly (*p*-value: 0.176)

The estimated mean BLS score of the students who had prepared themselves with online training was 7.60 points (95% CI: 6.76 to 8.44) and the score of the students of the control group was 6.81 (95% CI: 5.97 to 7.65) adjusted for *BLS training* and *class*. The maximum score was 10 points. The estimated mean difference between the groups was 0.79 (95% CI: -0.40 to 1.97) and therefore not significantly different (*p*-value: 0.176) (Fig. 3).

The variables *cluster* (*p* = 0.049) and *BLS-training status* (independent from the study) (*p* < 0.001) had a significant impact on mean BLS scores. Students who had previously participated in a BLS training had significantly higher mean scores in the BLS exam compared to students who had never participated in a BLS training.

### Secondary outcomes

#### Pass-rates on item level

**Fig. 4:**
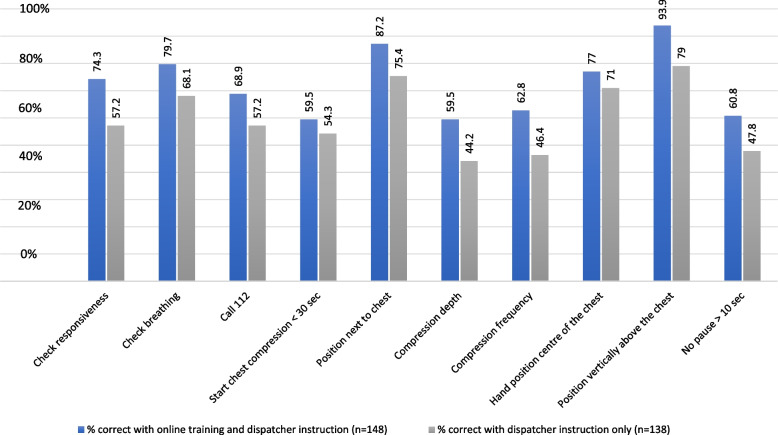
Calculated pass-rates on item level for the intervention and control group

Figure 4 illustrates the comparison of pass rates for each assessment item between groups that have undergone online training and those that have not. It appears that online training may be associated with higher pass rates, however, the difference is not statistically significant. The mixed logistic regression demonstrated a significant effect of the intervention on the chance to pass in one item. For the assessment item “vertically above the chest”, the odds ratio (OR) of correct performance was 4.99 (95% Cl: 1.46 to 17.12), indicating a higher likelihood of correct performance in the group that had undergone online training, compared to the students who did not receive online training. This difference was statistically significant with a *p*-value of 0.011. For compression depth a significant effect was just missed (OR 2.05, 95% CI: 0.97 to 4.33, *p*-value: 0.061). The odds ratio for various items (e.g., check responsiveness, check breathing…) were close to two but missed significance.

### Pass-rates

Preparation with online training before dispatcher assistance did not significantly improve the chance to pass the BLS exam compared to dispatcher assistance only (OR 1.56, 95% CI: 0.53 to 4.56, *p* = 0.416).

## Discussion

This study demonstrated that a 30-min online Basic Life Support (BLS) training, included in the biology lessons of high school students within two weeks prior to a simulated cardiac arrest scenario with dispatcher assistance, did not result in a significant enhancement in BLS performance. The intervention has a positive effect on BLS performance, but the effect-size was overestimated in the initial sample-size calculation. The potential of online training aligns with existing evidence [21, 22]. In our view, however, there are three pertinent aspects to consider when evaluating the impact of online training on practical BLS performance in this study.

First: In the assessed population of high school students about 30% of the students had participated in a face-to-face BLS training recently. Therefore, BLS skills were good, and so the scope for improvement is small. With less educated participants the effect of the online training may be more pronounced.

Second: The intervention had a positive effect on all items, though the effect size varied depending on the item. For certain technical aspects, such as the vertical position above the patient, compression depth, and initial check, the odds ratios were approximately two. However, the effect size was notably lower for the initiation of chest compression within the first 30 s and the emergency call. The diminished effect of the intervention on the initiation of CPR may derive from a testing effect, as neither group had prior experience with scenario testing. Additionally, the dispatcher instruction sequence did not schedule the start of chest compression within the first 30 s, leading to a construct-irrelevant variance [23].

Third: Retrospectively, the quality of the online course may have been insufficient or inconvenient for performing Basic Life Support (BLS) with dispatcher assistance. However, this inadequacy does not stem from poor conception or implementation of the online learning opportunity. Evidence-based elements, assumed to significantly impact learning, were duly considered. For instance, the course emphasized the relevance of skills for each individual, and the cognitive load was systematically organized and incrementally increased [24]. It is also important to note that there is limited research on factors influencing the effectiveness of dispatcher-assisted telephone instruction on CPR and how to prepare bystanders for dispatcher assistance [25, 26].

In our opinion, the primary reason why online-only education appears less effective than face-to-face education is the absence of practical training. Studies that support the effectiveness of self-regulated digital training invariably include hands-on practical training with manikins [22]. Referring to our data from another study in the same setting, we can affirm that a 45-min face-to-face training resulted in superior BLS performance in a comparable group of students. The mean scores of the students who participated in 45 min face-to-face practical BLS training were 9.34 (95% CI: 8.86 to 9.82), compared to 7.60 points (intervention group) and 6.81 points (control group) in this study [22, 27].

The impact of the scheduled online training, which resulted in a 0.8-point improvement in BLS performance, may appear modest. However, it aligns with the trend of skill decline observed within a year without training [20].

Considering The Lancet Commission’s goal to reduce the global burden of sudden cardiac death, we recognize that the first three links in the chain of survival—activation of emergency response, high-quality CPR, and defibrillation—have the most significant impact on survival rates [28]. Even small interventions in these areas can lead to substantial differences.

To enhance the effectiveness of dispatcher assistance, further research is strongly encouraged. Leveraging the widespread reach, reliability, and cost-effectiveness of digital education, along with recognizing the profound impact of dispatcher assistance on both survival rates and bystander well-being, we recommend aligning educational efforts with local dispatch practices and terminology.

### Strengths

The study has a low risk of bias according to the Cochrane List of bias for randomised trials, concerning randomisation, blinding and analysis of data. The randomisation and allocation of the students was done quasi randomly. Skills were assessed with an objective structured assessment with a high interrater reliability. The influences of the baseline differences between the student groups due to high cluster differences was respected by using a mixed-model analysis. All outcomes were analysed and reported as pre-specified in the study protocol. Blinding of the assessor was not broken. Even though this randomised controlled trial has a low risk of bias the study has some important limitations.

### Limitations

The study was underpowered. We did not reach the calculated sample size, because we could not recruit and assess 200 students in both study arms and the mean difference between the intervention and control was with about 0.8 points in the BLS exam lower than expected. Two classes allocated to the intervention did not participate in the assessment. We cannot estimate the effect of these dropouts and do not know the reason for not participating. Based on the observed mean difference the post-hoc sample size calculation adds up with 600 students per group to test a significant impact of the intervention.

We did not measure whether all students of the intervention received the intervention. We assume a high adherence of the teachers to facilitate the online training but did not capture user statistics.

The influence of the intervention on the ability to detect cardiac arrest and the willingness to perform BLS was not investigated. An impact of population wide online training on bystander CPR rates and survival can be assumed but the feasibility and efficacy of such an intervention needs to be investigated [29].

The results are closely linked to the local protocol of dispatcher assistance in use at this time [30]. In this protocol the sequence of the initial check and instruction cannot be cut short by using key words.

## Conclusion

Practical training in BLS is crucial for acquiring highly effective skills to treat cardiac arrest. Online training may have some positive impacts on the ability to recognise cardiac arrest and the quality of chest compression with dispatcher assistance. Therefore, online training could be one component in a set of interventions to establish a life-saving system. Beyond the already established elements in the system, such as creating awareness for the importance of bystander CPR, optimising the interplay between educational efforts and dispatcher assistance—for instance, by using the same keywords—could be a promising goal.

## Data Availability

Relevant data are included within the body of this manuscript. All raw and analysed data and materials are securely held on a password protected computer system in the Department of Anaesthesiology of the University Hospital Hamburg-Eppendorf (where the study was completed). The datasets are not publicly available to avoid backtracking and performance comparison to the participating schools but are available from the corresponding author on reasonable request.
